# Supplementary material for: Coherent two-dimensional electronic mass spectrometry
Source: Nat Commun. 2018 Jun 28;9:2519. doi: 10.1038/s41467-018-04927-w (PMC6023891; doi:10.1038/s41467-018-04927-w)
Supplement: Supplementary file 1 — Supplementary Information [file 41467_2018_4927_MOESM1_ESM.pdf]

Supplementary Information: Coherent two-dimensional electronic mass spectrometry

Sebastian Roeding[1] and Tobias Brixner[1,*]

[1]*Institut für Physikalische und Theoretische Chemie, Universität Würzburg, Am Hubland, 97074 Würzburg, Germany*
[*]*Corresponding author: brixner@phys-chemie.uni-wuerzburg.de*

May 30, 2018

Supplementary Note 1: Power Dependence

Supplementary Figure 1(a) displays the ion yield power dependency using the UV pulse only. The linear fit yields a slope of 2.77 for the parent ion, indicating that ionization with the UV pulse occurs by two competing processes comprising two or three photons, respectively. The fragment power dependency yields a slope of 2.70 meaning that it requires a similar amount of energy to generate the fragment ion via the ionization of a dissociation product as discussed in the main text.

The power dependence of the pump–probe signal of NO_2^+ is shown for the two-color experiment in Supplementary Figure 1(b). Power variation is carried out by rotating a half-wave plate in front of a polarizer and the pump–probe signal is extracted by a chopper sequence as described in the main text. Power-law fits (black lines) to the experimental data show that the pump–probe signal at a delay of $\Delta = 250$ fs requires absorption of two UV photons (blue circles) and one photon of the visible pulse (green circles), corroborating the conclusions of the pump–probe experiments using a UV pump and a visible probe pulse. As can be inferred from the transients shown in Supplementary Figure 5 of the main text, the fragment ion signal is much weaker compared to the parent ion signal at positive delay time. Thus, it is stronger affected by experimental noise, which especially in low-power conditions would obscure the power dependency. Therefore, we did not obtain the number of photons contributing to the NO^+ transient ion signal, as the range of the signal was much too small to yield a reliable fit.

Supplementary Figure 1(c) shows the power dependence of NO_2^+ and NO^+ ion yields for the single-color experiment using one visible pulse only. The fits (black lines) demonstrate that it requires four visible photons to generate NO_2^+ ions (blue circles) and five photons for the NO^+ fragment ions (green circles). This is in line with previous observations in the literature, where the dominant ionization

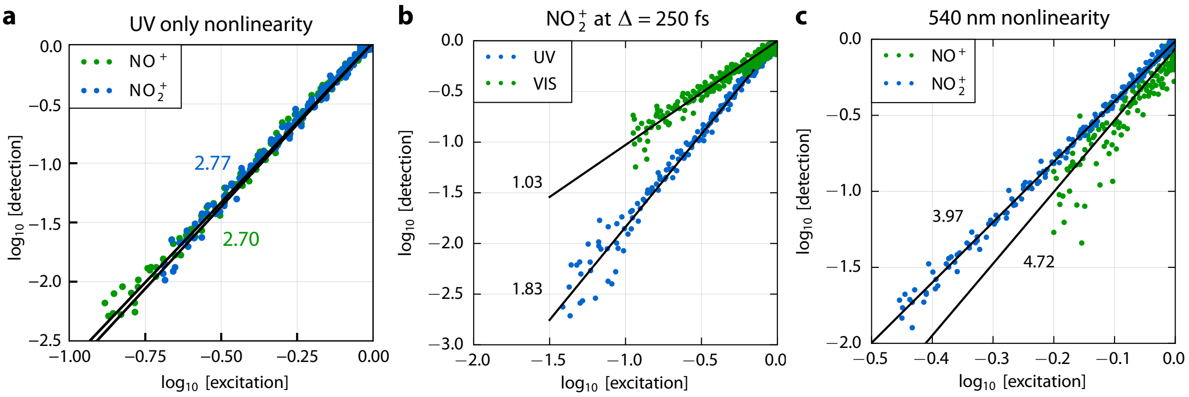

Supplementary Figure 1: Signal nonlinearity. Data have been normalized prior to analysis. (a) NO_2^+ (blue) and NO^+ (green) signal using the 267 nm pulse only with corresponding fits (black) and power-law exponents. (b) NO_2^+ pump–probe signal at a delay time of $\Delta = 250$ fs under variation of the 267 nm pump (blue) and the 540 nm probe (green) intensity. Fits are shown as solid lines with power-law exponents indicated. (c) NO_2^+ (blue) and NO^+ (green) signal using the 540 nm pulse only with corresponding fits (black) and power-law exponents.

pathway at wavelengths larger than 500 nm has been attributed to a (1+2+1) resonance-enhanced multiphoton-ionization processes [1]. In particular, the NO_2 molecule is resonant at the one-photon level, exciting the molecule into the $^2A_1/^2B_2$ manifold. Furthermore, at the level of three photons, the 3s Rydberg states at a total energy of about 7 eV come into resonance and can be used as stepping states towards ionization. The vertical ionization potential of NO_2 at the equilibrium geometry of the ground state is 11.23 eV [2] that cannot be overcome by four photons in the spectral region used here. However, at the resonance level of the third photon, the equilibrium geometry of the molecule changes from a bent ground-state configuration to a linear configuration, which is identical to the equilibrium configuration of the ionic ground state at an energy 9.586 eV [2]. At this geometry the energy of four photons of our spectrum ranging from 510 nm to 570 nm is just below the ionization potential into the ionic ground state.

Supplementary Note 2: Multiphoton Ionization 2D spectroscopy

In the main text, we show the rephasing 2D spectra of NO_2^+ and NO^+ for the experiment using only the visible pulses and no UV pump pulse. In Supplementary Figure 2 we additionally show the corresponding non-rephasing ($\alpha = 1$, $\beta = -1$, $\gamma = 1$, $\delta = -1$) contributions for the parent ion [Supplementary Figure 2(a)] and the NO^+ fragment [Supplementary Figure 2(b)]. Furthermore, we show in Supplementary Figure 2(c) Feynman diagrams with up to 10 interactions. They survive the phase-cycling scheme applied in this experiment because of aliasing and therefore potentially

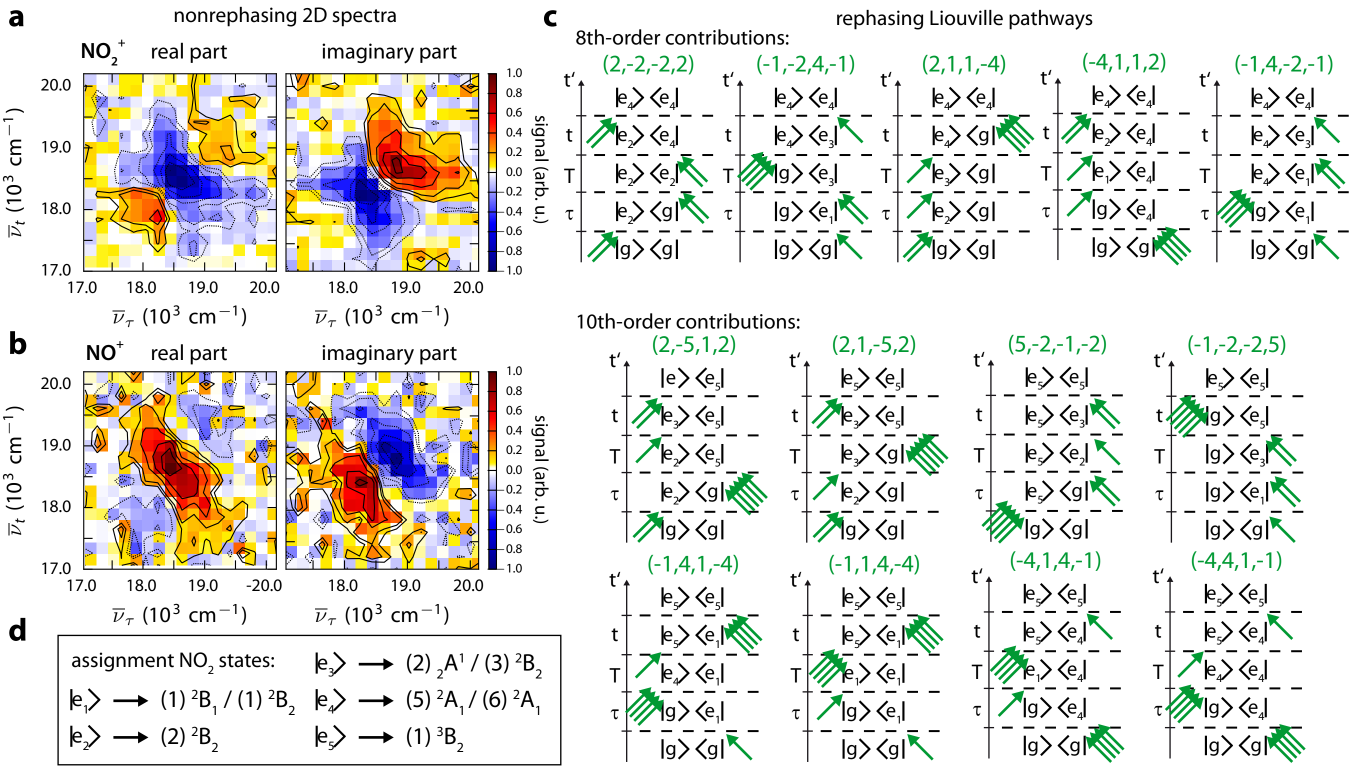

Supplementary Figure 2: 2D spectra using only the visible pulses. (a) Real part (left) and imaginary part (right) of the non-rephasing signal for the parent ion. (b) Real part (left) and imaginary part (right) of the non-rephasing signal for the NO^+ fragment. (c) Possible aliased Liouville pathways of higher nonlinearity that are contributing to the rephasing fourth-order signal shown in Figs. 2(a) and (c) of the main text and cannot be discriminated with the $1 \times 3 \times 3 \times 3$ phase-cycling scheme used here. Higher-step phase cycling can in principle be employed to discriminate between some of the pathways. (d) Assignment of possible NO_2 states to the general labels in (c).

contribute to the detected signal. All diagrams feature multiple quantum coherences of different kind that can generally be discriminated when the measurement is performed in the laboratory frame. As we have performed the measurement in the rotating frame, all contributions that are otherwise distributed over frequency space overlap and appear centered at the reference frequency. Supplementary Figure 2(d) shows possible assignments of the general energy-level place holders of the Feynman diagrams of Supplementary Figure 2(c) to specific states in the example of NO_2.

In this experiment, the most economic phase-cycling scheme was chosen that allows us to resolve the oscillation of a single-quantum coherence signal as a function of the relative phase between two consecutive pulses in a four-pulse experiment without aliasing. For higher-order nonlinear processes as shown in Supplementary Figure 2(c), double (triple) quantum coherences are excited that oscillate rapidly as a function of the relative phase between two pulses and are thus undersampled by the

discrete phase increments chosen in our case. Hence, analogous to an ordinary Fourier transformation, aliased signals with rapid oscillations overlap with signals of lower frequency. The question remains how these highly nonlinear signals could be separated after all. Three approaches are conceivable. The first possible approach is to refrain from using phase cycling and instead to perform the measurement in the laboratory frame ($\gamma = 1$). In the case of 8th (10th) order contributions, 7 (9) time delays need to be scanned with a temporal resolution of about 1 fs in order to fully resolve all contributions, rendering this an extremely complicated experiment. The second option is to perform the measurement in a fully rotating frame and to use an appropriate phase-cycling scheme to uniquely discriminate all contributions. Aiming to resolve contributions where each of the 8 pulses only interacts once, a $1 \times 3 \times 3 \times 3 \times 3 \times 3 \times 3 \times 3$ phase-cycling scheme is necessary, similar to the case of four collinear laser pulses. Generally, an intermediate approach using a partly rotating frame (e.g., $0 < \gamma < 1$) for the 7 (9) time delays and a reduced phase-cycling scheme is also possible, combining both approaches.

Supplementary Note 3: Transient 2D spectroscopy

Supplementary Figure 3(a) displays the transient rephasing 2D spectrum of the NO_2^+ parent ion at a population time $T = 30$ fs. Despite the somewhat noisy appearance the peak along the diagonal can be clearly identified. In order to aid identification of essential peak features we apply a Gaussian filter to the raw rephasing time-domain data with a FWHM of 1.4 time pixels (corresponding to 14 fs), thereby removing high-frequency contributions originating from experimental noise while retaining the essential peak features. This yields the rephasing 2D spectrum as shown in Supplementary Figure 5(a) and in Supplementary Figure 3 of the main text. Such a procedure is justified in our case as the measurement has been performed in the rotating frame, shifting all frequencies to the origin of frequency space and allowing to remove high frequency components originating from experimental noise. For plotting, we add the rotating frame center frequency and display the spectra with laboratory-frame frequency axis.

Furthermore, in Supplementary Figure 3(b) we show the rephasing 2D spectrum as obtained by scanning the identical visible pulse sequence with a photodiode placed in the beam path outside the vacuum chamber. This measurement was performed immediately before the actual 2D experiment with no change in experimental settings. No residual rephasing signal can be identified, meaning that nonlinear contributions to the signal of Supplementary Figure 3(a) as introduced, e.g., by pulse shaper artifacts can be ruled out. Furthermore, we can exclude artifacts arising from stray-light contributions as the detection is based on non-optical signals. The 2D signal arises solely from the amplitude of the parent ion mass peak and its dependence on the parameters of the four-pulse sequence (time delays and phases). Supplementary Figure 3(b) displays Feynman diagrams in addition to the one shown in Supplementary Figure 3 of the main text. They can also contribute to the ($\alpha = -1$, $\beta = 1$, $\gamma = 1$, $\delta = -1$) rephasing signal.

As stated in the main text, the two pathways leading to the ionic continuum starting from the prepared 3d Rydberg states, direct ionization and absorption into auto-ionizing states can interfere

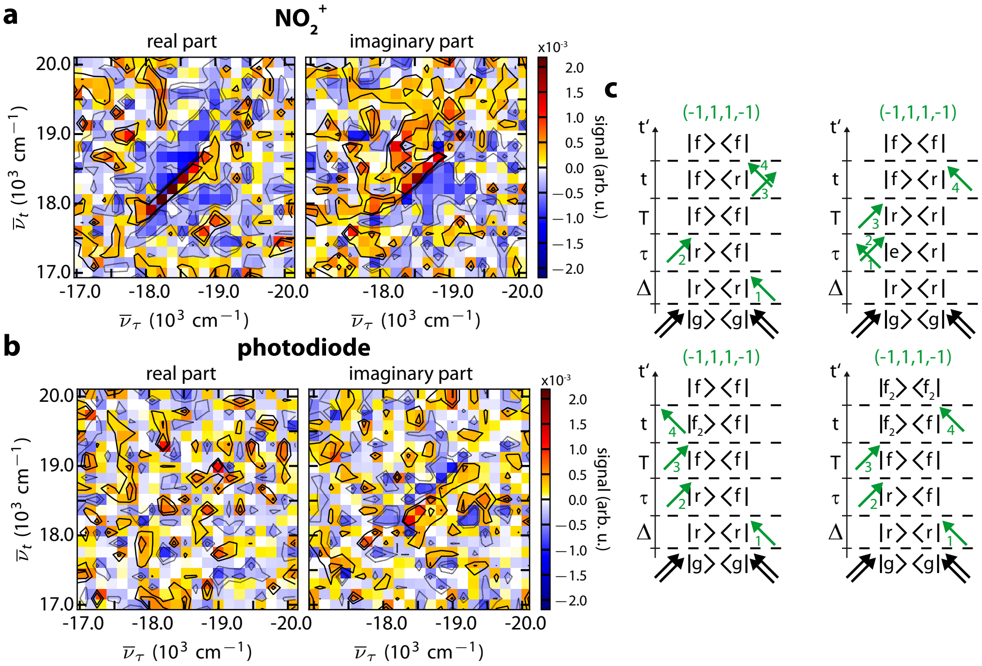

Supplementary Figure 3: Real and imaginary part of the rephasing 2D spectrum. (a) NO_2^+ parent ion, featuring a nonlinear signal elongated along the diagonal as discussed in the main text. (b) Photodiode signal, displaying zero signal and thus ruling out the possibility of pulse shaper artifacts contributing to the signal of (a). The photodiode has been placed at the laser focus position outside the chamber. Both measurements were conducted consecutively without any change in the setup.(c) Additional Feynman diagrams that are possible contributions to the ($\alpha = -1$, $\beta = 1$, $\gamma = 1$, $\delta = -1$) rephasing signal. In addition to the definition given in the main text, $|e\rangle$ indicates an excited state energetically below the Rydberg state, $|f_2\rangle$ a second, higher-lying final state.

giving rise to a Fano-type lineshape of the absorption profile [3].

The model of Supplementary Reference [4] requires not only the Fano q parameter describing the discrete–continuum coupling, but also a parameter Γ, that is given by

$$\Gamma = \frac{\gamma_\mathrm{e}}{\gamma_\mathrm{e} + \gamma} \qquad \in [0, 1] \,, \tag{1}$$

with γ_e denoting the dephasing of the coherences due to coupling to the continuum and γ denoting the pure dephasing due to the environment. Thus, Γ is a measure of the contribution of dissipation to the environment, with $\Gamma = 0$ ($\Gamma = 1$) indicating a relatively strong (weak) coupling to the bath. For a detailed derivation of analytical expressions for the rephasing and nonrephasing response functions see Supplementary Reference [4]. A large q parameter denotes weak coupling and a dominating transition to discrete auto-ionizing states. In contrast, small q denotes either a strong direct transition from the 3d Rydberg states to the ionic continuum or strong coupling via the auto-ionizing states. For

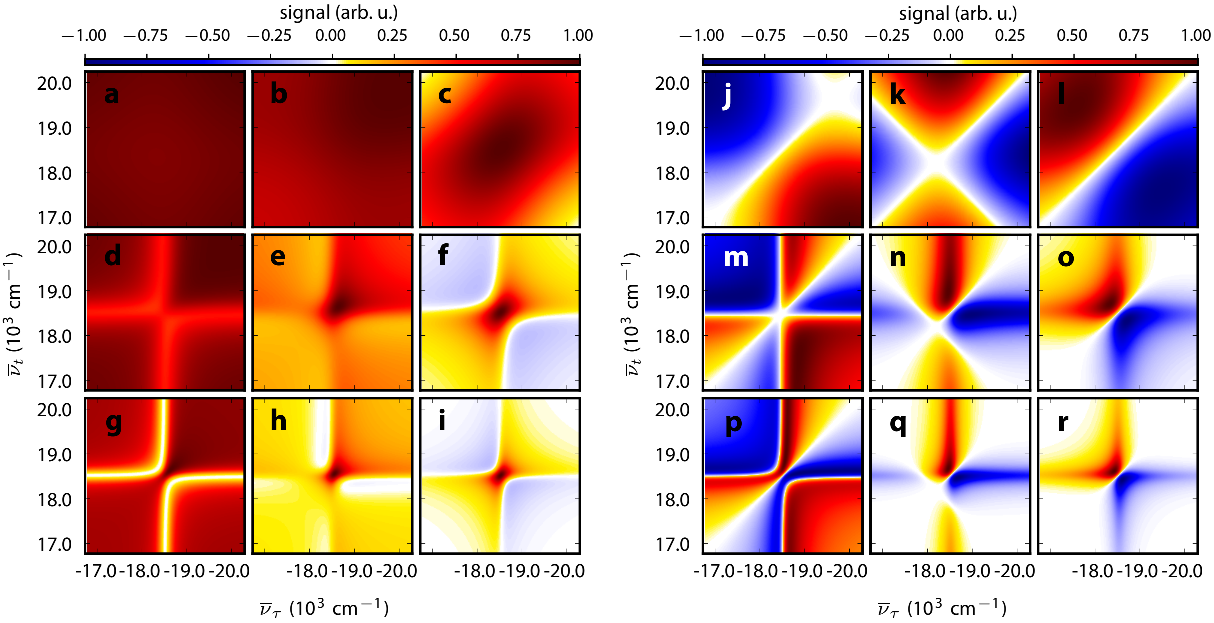

Supplementary Figure 4: Rephasing 2D spectrum of a Fano model. Calculated rephasing 2D spectrum using the 2D Fano model for different values of the Fano q parameter (varied for the individual plots along the vertical assembly direction) and the dissipation parameter Γ (along the horizontal direction). (a-i) Real part, (j-r) imaginary part: $\Gamma = 0.1, q = 0.1$ (a, j), $\Gamma = 0.1, q = 1.0$ (b, k), $\Gamma = 0.1, q = 10$ (c, l), $\Gamma = 0.5, q = 0.1$ (d, m), $\Gamma = 0.5, q = 1.0$ (e, n), $\Gamma = 0.5, q = 10$ (f, o), $\Gamma = 1.0, q = 0.1$ (g, p), $\Gamma = 1.0, q = 1.0$ (h, q), $\Gamma = 1.0, q = 10$ (i, r).

increasing q, the coupling to the continuum decreases, changing the asymmetric Fano lineshapes into Lorentzian-like lineshapes of discrete states.

Whereas q significantly influences the lineshape of the real part [Supplementary Figure 4(a-i)] and the imaginary part [Supplementary Figure 4(j-r)] of the rephasing signal, Γ scales the linewidth, depending on γ_e and γ. We have chosen $\gamma_\mathrm{e} = 160$ cm^{-1} for qualitative comparison of the influence of Γ and q on the 2D lineshape in Supplementary Figure 4.

Comparing the different lineshapes of the Fano model with our data [Supplementary Figure 5] we conclude that the lineshape observed in the experiment [Supplementary Figure 5(a)] has most similarity with a Fano model with a large q parameter ($q = 10$). The correspondence is most easily established when considering first the imaginary part of the signal. The imaginary part of the measured data [Supplementary Figure 5(a), right] has a node on the diagonal and negative (positive) signal below (above), which rules out Fano models with $q \leq 1$ [Supplementary Figure 4(j-r)]. Additionally, the real part is narrow and elongated along the diagonal and exhibits a change of sign along the cross-diagonal [Supplementary Figure 5(a)], favoring large q parameters.

From this observation, we infer an isolated sample with weak coupling of the 3d Rydberg states to the ionic continuum and thus a dominating transition to discrete auto-ionizing states. For further

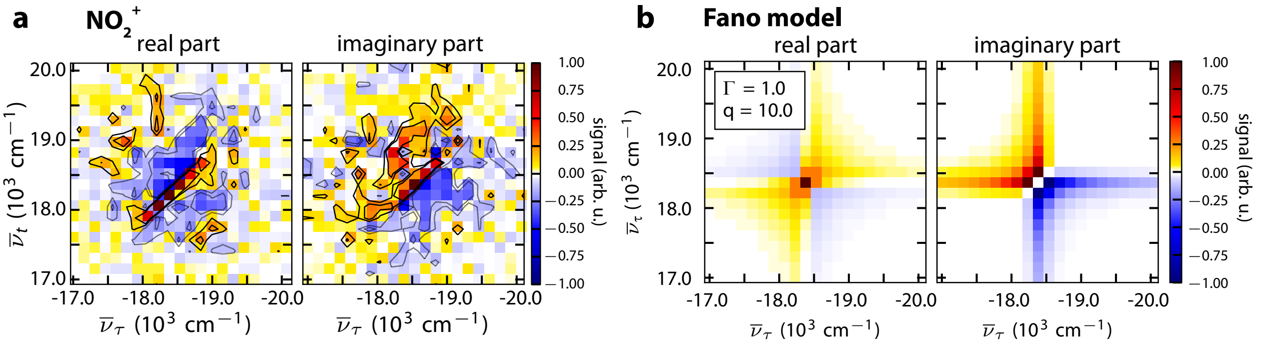

Supplementary Figure 5: Comparison of experimental rephasing 2D spectrum and Fano model. (a) Experimental rephasing 2D spectrum of the NO_2^+ parent ion. (b) Simulated Fano 2D spectrum of best agreement with the experimental data. The frequency resolution is equal to the experiment. Here, $\Gamma = 1$ and $q = 10$, indicating a transition to a discrete state and no dissipation to the environment.

investigation, an increased spectral resolution is beneficial. However, this comparison already demonstrates that our new method can be a valuable tool for the investigation of Fano-type resonances.

Supplementary Note 4: Pump–probe mass spectrometry

As stated in the main text, we recorded conventional pump–probe data over a larger range of delay times using a conventional mechanical delay stage. Here, we would like to give an extended discussion of the different signal contributions.

The model used to fit the pump–probe data is chosen in such a way that it results in a correct fit over the full time-domain range with the smallest possible number of parameters. The main intention was to describe the data for positive delay times as accurately as possible, as the transient 2D experiment is also performed at a positive delay between the UV and the visible pulses. We varied the number of parameters used in the fitting model to describe the dynamics at positive and negative delay times and only obtained a converging fit for the presented configuration comprising in total six decay constants (two at negative and four at positive delay times). The parameter uncertainties result from the square root of the diagonal entries of the covariance matrix $\mathbf{C}$ that is calculated via

$$\mathbf{C} = \left[\mathbf{J} \cdot \mathbf{J}^{\mathrm{T}}\right]^{-1} \sigma \tag{2}$$

with the mean-squared error σ of the fit and the Jacobian $\mathbf{J}$ and the transposed matrix $\mathbf{J}^{\mathrm{T}}$ that is returned by the applied Levenberg-Marquardt solver.

We fit both traces simultaneously using a parallel kinetic rate model [5, 6] resulting for positive delay times (for which the UV acts as a pump) in a decay with time constants of $\tau_1^+ = (60 \pm 10)$ fs, $\tau_2^+ = (264 \pm 47)$ fs (red line), $\tau_3^+ = (3222 \pm 589)$ fs, and a slow decay of several hundred picoseconds for the fragment trace. For negative delay times, a decay with $\tau_1^+ = (141 \pm 106)$ fs and an additional persistent offset is observed for the fragment trace. All signal contributions in

Supplementary Figure 5(a) are convoluted with a Gaussian instrument response function centered at time zero and a FWHM of (100 ± 2) fs as obtained from the fit. The instrument response function is added as a separate contribution to account for the ion yield during pulse overlap and only contributes to the parent ion with non-negligible amplitude (blue line).

We start with a discussion of the data for the negative delay times where the visible laser pulse acts as the pump. In contrast to the single ultrafast decay for NO_2^+, the NO^+ fragment is dominated by an initial decay with a time constant of (141 ± 106) fs and a persistent signal for up to -100 ps (long-time data measured in a separate experiment). The results can be rationalized by considering the complex absorption profile of NO_2^+ between 250 nm and 650 nm that is caused by mixing of several excited-state potential-energy surfaces [2]. The threshold for the first dissociation limit of NO_2 in this excited-state manifold lies at an energy of 3.12 eV [2]. A 540 nm pump photon is equivalent to an energy of 2.30 eV and therefore leads to an excitation below the dissociation limit into the mixed $^2A_1/^2B_2$ band. Vredenborg et al. [7] observed a contribution with a decay timescale of less than 200 fs after the absorption of one 400 nm pump photon (3.10 eV) in (NO_2^+, e^-) coincidence experiments. They attributed the timescale of this contribution to a spreading of the prepared wavepacket on the excited state which reduces the cation yield of the subsequent ionization pulse. If no dissociation takes place, the lifetime of the prepared excited states is estimated to be on the order of μs in collision-free environments [2]. This is most likely the reason for the persistent offset in the pump–probe signal at negative delay times. For positive delay times (UV acts as pump), both fragments show a fast initial relaxation with a time constant of $\tau_1^+ = (60 \pm 10)$ fs and a subsequent decay with $\tau_2^+ = (265 \pm 47)$ fs (red line) and $\tau_3^+ = (3222 \pm 589)$ fs. Whereas the parent ion completely decays to zero for $\Delta > 10$ ps, the fragment ion exhibits a remaining signal for larger pump–probe delays with a decay time exceeding the scanning range of the measurement. Previous experiments using 267 nm pump and 400 nm probe pulses identified several excitation pathways using velocity-map imaging [7–12] and coincidence detection [13]. One major ionization pathway contributing to the pump–probe signal of the parent ion was identified as the excitation to the 3d Rydberg states of NO_2 at an energy of 9.2 eV by the absorption of 2 (3) 267 nm (400 nm) photons. In C_{2v} symmetry they correlate with the 5 $^2A_1$ and 6 $^2A_1$ states [2] [Supplementary Figure 5(b)]. Already in earlier fluorescence depletion experiments [14] these Rydberg states were shown to dissociate on a timescale of less than 500 fs and form excited NO in the $A^2\Sigma^+$ state. In addition, a contribution from long-lived Rydberg states at these energy levels has also been observed [7,11]. Hence, we attribute the observed dynamics of the parent ion, in particular the 265 fs decay, to the absorption of two 267 nm pump photons and subsequent dissociation of highly-excited NO_2, as shown in Supplementary Figure 5(b). The probe step ionizes the molecule by the absorption of one 540 nm photon, leading to the observed ion signal. Accordingly, we assign the long-lived contribution in the NO^+ signal to the product of neutral NO_2 dissociation and subsequent ionization by multiple photons of the probe pulse. Moreover, the initial fast relaxation dynamics observed for the parent ion is also present in the fragment trace. This signal cannot originate as a direct product from neutral NO_2 dissociation and must result from the final NO_2 state that is reached from the 3d Rydberg states after absorption of the probe photon.

Recent experiments [15] discussed the role of high-lying Rydberg states in photoionization of NO_2

Time constant (this work) [fs]	Assignment	Literature
$\tau_1^- = 141 \pm 106$	$^2B_2$ Wavepacket spreading	< 200 fs [7]
$\tau_2^- \to \infty$ (offset)	$^2B_2$ Excited-state lifetime	> 10 μs [2,23]
$\tau_1^+ = 60 \pm 10$	3d Rydberg states lifetime	no clear assignment found
$\tau_2^+ = 265 \pm 47$	3d Rydberg states lifetime	< 500 fs [7,9–12,14,24–26]
$\tau_3^+ = 3222 \pm 589$	Long-lived Rydberg state	proposed in [7,11]
$\tau_4^+ \to \infty$ (persistent fragment)	Dissociation product	

Supplementary Table 1: Observed time constants and literature assignment. Time constants with a positive (negative) superscript correspond to the UV (visible) pulse acting as the pump.

and highlighted the aspect of (NO^+, O^-) ion pair formation at energies of about 11.5 eV in addition to auto-ionization [16, 17]. This energetic region is accessible in our experiment by (2+1)-excitation (11.58 eV), which is why we assign the origin of the NO^+ signal at short delay times to the ion pair-formation state. Consequently, the parent ion is conjectured to be generated via absorption to these high-lying Rydberg states and subsequent auto-ionization [18–22]. In Supplementary Table 1 we summarize the observed timescales and assignments of different processes.

Supplementary References

[1] Morrison, R. J. S., Rockney, B. H. & Grant, E. R. Multiphoton ionization of NO_2: Spectroscopy and dynamics. *J. Chem. Phys.* **75**, 2643–2651 (1981).

[2] Wilkinson, I. & Whitaker, B. J. Some remarks on the photodynamics of NO_2. *Annu. Rep. Prog. Chem., Sect. C: Phys. Chem.* **106**, 274–304 (2010).

[3] Fano, U. Effects of configuration interaction on intensities and phase shifts. *Phys. Rev.* **124**, 1866–1878 (1961).

[4] Finkelstein-Shapiro, D., Poulsen, F., Pullerits, T. & Hansen, T. Coherent two-dimensional spectroscopy of a Fano model. *Phys. Rev. B* **94**, 205137 (2016).

[5] Zewail, A. H. & Pedersen, S. Femtosecond real time probing of reactions XXII: kinetic description of probe absorption, fluorescence depletion and mass spectrometry. *Mol. Phys.* **89**, 1455–1502 (1996).

[6] van Stokkum, I. H. M., Larsen, D. S. & van Grondelle, R. Global and target analysis of time-resolved spectra. *Biochim. Biophys. Acta, Bioenerg.* **1657**, 82–104 (2004).

[7] Vredenborg, A., Roeterdink, W. G. & Janssen, M. H. M. Femtosecond time-resolved photoelectron-photoion coincidence imaging of multiphoton multichannel photodynamics in NO_2. *J. Chem. Phys.* **128**, 204311 (2008).

[8] Davies, J. A., LeClaire, J. E., Continetti, R. E. & Hayden, C. C. Femtosecond time-resolved photoelectron–photoion coincidence imaging studies of dissociation dynamics. *J. Chem. Phys.* **111**, 1–4 (1999).

[9] Eppink, A. T. J. B. *et al.* Dissociative multiphoton ionization of NO_2 studied by time-resolved imaging. *J. Chem. Phys.* **121**, 7776–7783 (2004).

[10] Form, N. T., Whitaker, B. J., Poisson, L. & Soep, B. Time-resolved photoion and photoelectron imaging of NO_2. *Phys. Chem. Chem. Phys.* **8**, 2925–2932 (2006).

[11] Cireasa, R., Hamard, J.-B., Maury, C. & Blanchet, V. Imaging fast relaxation dynamics of NO_2. *Phys. Scr.* **80**, 048106 (2009).

[12] Irimia, D., Petsalakis, I. D., Theodorakopoulos, G. & Janssen, M. H. M. Coherent oscillatory femtosecond dynamics in multichannel photodynamics of NO_2 studied by spatially masked electron imaging. *J. Phys. Chem. A* **114**, 3157–3166 (2010).

[13] Forbes, R., Boguslavskiy, A. E., Wilkinson, I., Underwood, J. G. & Stolow, A. Excited state wavepacket dynamics in no_2 probed by strong-field ionization. *J. Chem. Phys.* **147**, 054305 (2017).

[14] López-Martens, R. B., Schmidt, T. W. & Roberts, G. Femtosecond fluorescence depletion spectroscopy of NO_2 multiphoton dissociation dynamics. *J. Chem. Phys.* **111**, 7183–7186 (1999).

[15] Poullain, S. M. *et al.* The role of Rydberg states in photoionization of NO_2 and (NO^+, O^-) ion pair formation induced by one VUV photon. *J. Chem. Phys.* **139**, 044311 (2013).

[16] Elkharrat, C. *et al.* Ion pair formation in multiphoton excitation of NO_2 using linearly and circularly polarized femtosecond light pulses: kinetic energy distribution and fragment recoil anisotropy. *J. Phys. Chem. A* **114**, 9902–9918 (2010).

[17] Poullain, S. M. *et al.* Recoil frame photoemission in multiphoton ionization of small polyatomic molecules: photodynamics of NO_2 probed by 400 nm fs pulses. *J. Phys. B: At. Mol. Opt. Phys.* **47**, 124024 (2014).

[18] Bell, P., Aguirre, F., Grant, E. R. & Pratt, S. T. Mode-dependent vibrational autoionization of NO_2. *J. Chem. Phys.* **119**, 10146–10157 (2003).

[19] Bell, P., Aguirre, F., Grant, E. R. & Pratt, S. T. Mode dependent vibrational autoionization of Rydberg states of NO_2. II. Comparing the symmetric stretching and bending vibrations. *J. Chem. Phys.* **120**, 2667–2676 (2004).

[20] Bell, P., Aguirre, F., Grant, E. R. & Pratt, S. T. State-selective production of vibrationally excited NO_2^+ by double-resonant photoionization. *J. Phys. Chem. A* **108**, 9645–9651 (2004).

[21] Pratt, S. Vibrational autoionization in polyatomic molecules. *Annu. Rev. Phys. Chem.* **56**, 281–308 (2005).

[22] Jungen, C. & Pratt, S. T. Renner–Teller interactions in the vibrational autoionization of polyatomic molecules. *J. Chem. Phys.* **129**, 164310 (2008).

[23] Santoro, F. & Petrongolo, C. Nonadiabatic radiative lifetimes and fluorescence spectra of NO_2. *J. Chem. Phys.* **111**, 9651–9657 (1999).

[24] Schmidt, T. W., López-Martens, R. B. & Roberts, G. Intense-field modulation of NO_2 multiphoton dissociation dynamics. *J. Chem. Phys.* **121**, 4133–4142 (2004).

[25] Liu, B., Zhu, J., Wang, B., Wang, Y. & Wang, L. Time-resolved dynamics of NO_2 in its conical intersection region. *J. Phys. Chem. A* **113**, 13839–13844 (2009).

[26] Hamard, J. B., Cireasa, R., Chatel, B., Blanchet, V. & Whitaker, B. J. Quantum interference in NO_2. *J. Phys. Chem. A* **114**, 3167–3175 (2010).
